# Association of the insulin-like growth factor binding protein 3 (IGFBP-3) polymorphism with longevity in Chinese nonagenarians and centenarians

**DOI:** 10.18632/aging.100703

**Published:** 2014-12-02

**Authors:** Yong-Han He, Xiang Lu, Li-Qin Yang, Liang-You Xu, Qing-Peng Kong

**Affiliations:** ^1^ State Key Laboratory of Genetic Resources and Evolution, Kunming Institute of Zoology, the Chinese Academy of Sciences, Kunming 650223, China; ^2^ KIZ/CUHK Joint Laboratory of Bioresources and Molecular Research in Common Diseases, Kunming 650223, China; ^3^ Dujiangyan Longevity Research Centre, Dujiangyan 611830, China

**Keywords:** insulin-like growth factor binding protein 3, longevity, single nucleotide polymorphism

## Abstract

Human lifespan is determined greatly by genetic factors and some investigations have identified putative genes implicated in human longevity. Although some genetic loci have been associated with longevity, most of them are difficult to replicate due to ethnic differences. In this study, we analyzed the association of 18 reported gene single nucleotide polymorphisms (SNPs) with longevity in 1075 samples consisting of 567 nonagenarians/centenarians and 508 younger controls using the GenomeLab SNPstream Genotyping System. Our results confirm the association of the forkhead box O3 (*FOXO3*) variant (rs13217795) and the ATM serine/threonine kinase (*ATM*) variant (rs189037) genotypes with longevity (p=0.0075 and p=0.026, using the codominant model and recessive model, respectively). Of note is that we first revealed the association of insulin-like growth factor binding protein 3 (*IGFBP-3*) gene polymorphism rs11977526 with longevity in Chinese nonagenarians/centenarians (p=0.033 using the dominant model and p=0.035 using the overdominant model). The FOXO3 and IGFBP-3 form important parts of the insulin/insulin-like growth factor-1 signaling pathway (IGF-1) implicated in human longevity, and the ATM gene is involved in sensing DNA damage and reducing oxidative stress, therefore our results highlight the important roles of insulin pathway and oxidative stress in the longevity in the Chinese population.

## INTRODUCTION

Human life span is influenced by multiple determinants, including various environmental and genetic factors. Though the non-genetic factors, such as diet, health habits, physical activity, and psychosocial factors are important, genetic factors have been shown to contribute to human life span by approximately 25% [1]. Interestingly, the heritability of longevity increases with greater age with the estimated heritability of living to at least 100 was 0.33 in women and 0.48 in men [2].

The mechanisms influencing lifespan have been widely investigated in various model organisms, such as *Caenorhabditis* elegans, *Saccharomyces cerevisiae*, and *Drosophila melanogaster*, and hundreds of genetic variants causing life extension have been identified [3-5], such as apolipoprotein E (*APOE*), forkhead box O3A (*FOXO3A*), cholesterylester transfer protein (*CETP*), exonuclease 1 (*EXO1*), etc. [6]. Of the candidate genes, variants in *APOE* and *FOXO3A* have been most consistently replicated in human populations while the others are difficult to validate in different populations. This could be due to the great differences in allele and genotype frequencies in the studied polymorphisms among ethnicities [7, 8]. Thus, it is highly desirable to conduct large-scale studies with adequate replication to identify variants that are likely to exert an effect on life span.

In this study, we collected 18 longevity-associated variants and investigated their associations with longevity in 1075 samples consisting of 567 nonagenarians/centenarians and 508 younger controls. As a result, our data confirms the reported associations of the *FOXO3* variant rs13217795 and the ATM serine/threonine kinase (*ATM*) variant rs189037 with longevity. In addition, we found a significant association of the insulin-like growth factor binding protein 3 (*IGFBP-3*) gene polymorphism rs11977526 with longevity, which has never been reported in the Chinese population.

## RESULTS AND DISCUSSION

In this study, we analyzed 18 reported longevity-associated polymorphisms in the longevity subjects and their matched controls. Genotypic distributions of all single nucleotide polymorphisms (SNPs) in the controls were in agreement with the Hardy–Weinberg Equilibrium (HWE) (all p values>0.05, Table 1). As shown in Table 1, the rs13217795 (*p*=0.016) and rs189037 (*p*=0.042) were identified to have differed allelic frequencies between the two groups. The polymorphism rs11977526 had marginal significance (*p*=0.064) in allelic frequency. The other variants (rs2717536, rs2153960, rs1377638, rs10069397, rs1245541, rs2244621, rs11977526, rs1063192, rs579327, rs1455311, rs2219078, rs2755213, rs12629971, rs1003533, rs189037, rs1442709 and rs6817112) did not show any significant difference between the two groups (all p values>0.05, Table 1). To minimize the bias caused by different ages between the control and longevity subject, we further compared the allele frequencies of SNPs to that in the general Chinese Han population retrieved from the available databases (HapMap Projects and 1000 Genomes Project), or published literatures. Consistently, the SNPs rs11977526, rs13217795 and rs189037 were shown to be significantly associated longevity (*p*=0.008, 0.002 and 0.009, respectively) (Supplemental Table 1). The genotypic frequencies and associations of SNPs with longevity are shown in Table 2. Consistent with the allelic association, the rs13217795 had a significant association with longevity either in the codominant model (minor genotype C/C vs. major genotype T/T, OR=0.50, 95% CI=0.31-0.79, p=0.0075) or in the recessive model (minor genotype C/C vs. T/T-T/C genotypes, OR=0.50, 95% CI=0.32-0.78, p=0.0018). For the SNP rs189037, the significance was marginal in the codominant model (minor genotype T/T vs. major genotype C/C, OR=1.50, 95% CI=1.04-2.16, p=0.076) while was significant in the recessive model (minor genotype T/T vs. C/C-T/C genotypes, OR=1.44, 95% CI=1.04-1.99, p=0.026). For the SNP rs11977526, although the allelic association was just marginal, the genotypes were found to differently distributed between the longevity and control groups in the dominant model (T/C-C/C vs. major genotype T/T, OR=0.76, 95% CI=0.58-0.98, p=0.033) and in the overdominant model (T/C vs. T/T-C/C genotypes, OR=0.75, 95% CI=0.57-0.98, p=0.035). However, the other 15 SNPs did not have any differences in the genotypic frequencies between the case and control groups (Supplemental Table 2). Above data suggest that the SNPs rs13217795, rs189037 and rs11977526 were associated with the longevity in the Chinese population.

**Table 1:** Allelic distributions of selected SNPs in the control and longevity subjects

	Control		Control number	Longevity		Longevity number	HWE for control	Allelic analysis			
	Major allele	Minor allele		Major allele	Minor allele			χ^2^	OR	% 95 CI	*p* Value
rs2717536	668 (0.71)	272 (0.29)	472	832 (0.73)	314 (0.27)	573	0.073	0.604	0.927	0.765-1.13	0.233
rs2153960	667 (0.71)	277 (0.29)	472	800 (0.7)	346 (0.3)	573	0.66	0.178	1.041	0.863-1.257	0.354
rs1377638	554 (0.59)	384 (0.41)	472	647 (0.56)	499 (0.44)	573	0.92	1.433	1.113	0.934-1.325	0.125
rs10069397	854 (0.91)	82 (0.09)	472	1056 (0.92)	90 (0.08)	573	0.24	0.56	0.888	0.649-1.213	0.252
rs1245541	806 (0.86)	132 (0.14)	472	980 (0.86)	166 (0.14)	573	0.45	0.072	1.034	0.808-1.324	0.419
rs2244621	479 (0.51)	463 (0.49)	472	568 (0.5)	578 (0.5)	573	0.85	0.324	1.053	0.886-1.251	0.294
rs11977526	751 (0.8)	185 (0.2)	472	887 (0.77)	259 (0.23)	573	0.31	2.469	1.185	0.959-1.466	0.064
rs1063192	751 (0.8)	185 (0.2)	472	942 (0.82)	204 (0.18)	573	0.77	1.308	0.879	0.705-1.096	0.139
rs579327	864 (0.92)	80 (0.08)	472	1033 (0.9)	113 (0.1)	573	0.13	1.186	1.181	0.875-1.595	0.155
rs1455311	779 (0.83)	165 (0.17)	472	958 (0.84)	188 (0.16	573	0.08	0.425	0.927	0.737-1.165	0.276
rs13217795	749 (0.74)	265 (0.26)	508	789 (0.7)	345 (0.3)	567	0.49	4.843	1.236	1.023-1.493	0.016
rs2219078	668 (0.66)	342 (0.34)	508	758 (0.67)	376 (0.33)	567	0.99	0.119	0.968	0.810-1.160	0.382
rs2755213	587 (0.58)	423 (0.42)	508	680 (0.6)	454 (0.4)	567	0.06	0.753	0.926	0.780-1.101	0.205
rs12629971	646 (0.64)	368 (0.36)	508	716 (0.63)	418 (0.37)	567	0.25	0.075	1.025	0.860-1.222	0.788
rs1003533	630 (0.62)	378 (0.38)	508	684 (0.61)	446 (0.39)	567	0.7	0.872	1.087	0.913-1.294	0.187
rs189037	551 (0.55)	455 (0.45)	508	662 (0.59)	468 (0.41)	567	0.79	3.153	0.856	0.721-1.016	0.042
rs1442709	562 (0.56)	450 (0.44)	508	633 (0.56)	501 (0.44)	567	0.15	0.018	0.988	0.833-1.172	0.464
rs6817112	651 (0.64)	359 (0.36)	508	734 (0.65)	400 (0.35)	567	0.7	0.017	0.988	0.828-1.180	0.466

OR, Odds ratio; HWE, Hardy–Weinberg Equilibrium; %95 CI, 95% confidence interval; P-values were adjusted by sex.

**Table 2:** Genotypic associations with longevity in Chinese nonagenarians and centenarians

SNP	Model	Genotype	Control	Longevity	OR (95% CI)	P-value *	AIC	BIC
rs13217795	Codominant	T/T	290 (51.1%)	273 (53.9%)	1	**0.0075**	1445.1	1465
		T/C	209 (36.9%)	203 (40%)	1.00 (0.77-1.29)		
		C/C	68 (12%)	31 (6.1%)	0.50 (0.31-0.79)		
	Dominant	T/T	290 (51.1%)	273 (53.9%)	1	0.29	1451.8	1466.7
		T/C-C/C	277 (48.9%)	234 (46.1%)	0.88 (0.69-1.12)			
	Recessive	T/T-T/C	499 (88%)	476 (93.9%)	1	**0.0018**	1443.1	1458.1
		C/C	68 (12%)	31 (6.1%)	0.50 (0.32-0.78)			
	Overdominant	T/T-C/C	358 (63.1%)	304 (60%)	1	0.46	1452.4	1467.3
		T/C	209 (36.9%)	203 (40%)	1.10 (0.86-1.41)			
	Log-additive	---	---	---	0.81 (0.67-0.98)	**0.03**	1448.2	1463.1
rs189037	Codominant	C/C	184 (32.6%)	149 (29.6%)	1	0.076	1441	1460.8
		T/C	294 (52%)	253 (50.3%)	1.07 (0.81-1.41)			
		T/T	87 (15.4%)	101 (20.1%)	1.50 (1.04-2.16)			
	Dominant	C/C	184 (32.6%)	149 (29.6%)	1	0.26	1442.8	1457.7
		T/C-T/T	381 (67.4%)	354 (70.4%)	1.16 (0.89-1.52)			
	Recessive	C/C-T/C	478 (84.6%)	402 (79.9%)	1	0.026	1439.2	1454.1
		T/T	87 (15.4%)	101 (20.1%)	1.44 (1.04-1.99)
	Overdominant	C/C-T/T	271 (48%)	250 (49.7%)	1	0.52	1443.7	1458.6
		T/C	294 (52%)	253 (50.3%)	0.92 (0.72-1.18)
	Log-additive	---	---	---	1.20 (1.00-1.44)	0.046	1440.1	1455
rs11977526	Codominant	T/T	342 (59.7%)	305 (65.2%)	1	0.094	1394	1413.8
		T/C	203 (35.4%)	141 (30.1%)	0.74 (0.57-0.97)			
		C/C	28 (4.9%)	22 (4.7%)	0.86 (0.47-1.55)			
	Dominant	T/T	342 (59.7%)	305 (65.2%)	1	0.033	1392.2	1407
		T/C-C/C	231 (40.3%)	163 (34.8%)	0.76 (0.58-0.98)
	Recessive	T/T-T/C	545 (95.1%)	446 (95.3%)	1	0.86	1396.7	1411.5
		C/C	28 (4.9%)	22 (4.7%)	0.95 (0.53-1.70)
	Overdominant	T/T-C/C	370 (64.6%)	327 (69.9%)	1	0.035	1392.3	1407.1
		T/C	203 (35.4%)	141 (30.1%)	0.75 (0.57-0.98)
	Log-additive	---	---	---	0.82 (0.66-1.01)	0.067	1393.4	1408.2

* P-values were adjusted by sex; OR, Odds ratio; %95 CI, 95% confidence interval; AIC, Akaike information criteria;

### BIC, Bayesian information criteria

As shown in Table 3, the rs13217795 and rs189037 was located in the intron region of *FOXO3A* and the promoter of *ATM* gene, respectively. *FOXO3A* gene is a critical downstream molecule of AKT1 in insulin/insulin-like growth factor (IGF) signaling pathways which has been well shown involved in the aging process from yeast to humans [9-11] and the AKT1 and mammalian target of rapamycin (mTOR) constitute two important parts of this pathway [12-16]. Genetic variations in *FOXO3A* have previously been associated with human longevity in Japanese, German, Italian and Chinese population-based studies [17-20]. Our results further confirm this association and indicate the possible involvement of IGF signaling pathways in determining human life span. The product of *ATM* gene is a critical protein in the p53 pathway and has been reported to be a nuclear protein involved in several signaling pathways, including DNA damage recognition, cell cycle control, and meiotic recombination [21]. In humans, patients with ATM gene mutations are characterized by insulin resistance, immunodeficiency, growth retardation, pigmentary abnormalities, progressive cerebellar degeneration, and increased susceptibility to cancer [22], suggesting ATM is likely to affect human lifespan. In fact, the *ATM* genetic variant rs189037 has been reported to be a functional locus associated with longevity in the Chinese population through affecting the mRNA expression of *ATM* [23]. This result was subsequently validated in an Italia population [24]. Our data further suggest the association of *ATM* variant rs189037 with longevity.

**Table 3:** Selected loci associated with longevity

SNP	SNP position	Band	Alleles	Nearest locus or loci
rs2717536	chr6:108974098	6q21	C/T	*FOXO3*
rs2153960	chr6:108988184	6q21	A/G	*FOXO3*
rs1377638	chr2:5293525	2p25.2	C/T	*SOX11*
rs10069397	chr5:65783709	5q12.3	C/T	*FLJ46010*
rs1245541	chr10:73849639	10q22.1	A/G	*ASCC1; SPOCK2*
rs2244621	chr11:64026219	11q13.1	C/T	*PLCB3*
rs11977526	chr7:46008110	7p12.3	A/G	*IGFBP-3*
rs1063192	chr9:22003367	9p21.3	A/G	*CDKN2B; CDKN2A*
rs579327	chr2:234768067	2q37.1	C/T	*MSL3L2; HJURP*
rs1455311	chr4:79964587	4q21.21	A/G	*PAQR3; NAA11*
rs13217795	chr6:108974098	6q21	C/T	*FOXO3*
rs2219078	chr2:108875198	2q12.3	A/G	*SULT1C3*
rs2755213	chr13:41146301	13q14.11	C/T	*FOXO1*
rs12629971	chr3:71783318	3p13	C/T	*EIF4E3*
rs1003533	chr5:131755651	5q31.1	C/T	*C5orf56*
rs189037	chr11:108093833	11q22.3	A/G	*ATM; NPAT*
rs1442709	chr11:20089978	11p15.1	A/G	*NAV2*
rs6817112	chr4:154080813	4q31.3	C/T	*TRIM2*

Of note is that we found an association between the SNP rs11977526 genotype and longevity either in the dominant model or in the overdominant model (Table 2). The rs11977526 was located in the *IGFBP3* region on chromosome 7p12.3 (Table 3), which is known to be associated with circulating IGFBP-3 levels [25]. IGFBP-3 is bound to about 90% of the circulating insulin-like growth factor-I (IGF-I) that exerts mitogenic and metabolic activities in the regulation of growth, survival and cell differentiation [26]. Albeit the rs11977526 is associated with circulating IGFBP-3 level, its association with longevity has not been reported until this study. Unfortunately, measurement of circulating IGFBP-3 levels in our samples depending on the rs11977526 genotypes have not been performed in this study, which might have forced the power of association which is weak but significant (p=0.033 and 0.035 in different models), and other large-scale studies in different ethnicities are needed to replicate this result in the future. In addition, functional evidence for the effect of this variant on life span are also helpful to understand the direct or indirect mechanisms that link the SNP with longevity.

By careful analysis we found that the above-described three SNPs associated with longevity are not independent from each other. For example, the FOXO3 (rs13217795) forms part of the IGF-1 signaling pathway, while the ATM (rs189037) is a critical protein in the p53 pathway involved in sensing DNA damage and reducing oxidative stress. The IGF-1 pathway highly interacts with the p53 pathway and both pathways constitutes important components involved in longevity [27-29].

In conclusion, our results confirm the reported association of the *FOXO3* and *ATM* gene polymorphisms (rs13217795 and rs189037, respectively) with longevity. More importantly, we first found a variant of *IGFBP-3* in the IGF-1 pathway, rs11977526, is associated with longevity in Chinese nonagenarians and centenarians. Due to the FOXO3 and IGFBP-3 are important molecules in the insulin/IGF-1 pathway, and ATM in the oxidative stress, our results highlight the important roles of insulin pathway and oxidative stress in the longevity in the Chinese population.

## METHODS

### Subjects

A total of 1075 samples consisting of 567 nonagenarians/centenarians (mean age 94.1) and 508 controls (mean age 51.7 years) were collected from Dujianyan district of Sichuan province of China in 2010 (Supplemental Table 3). All of the longevity subjects had no severe diseases according to their medical examinations [30]. The control subjects were all healthy with no severe medical history. Blood samples for DNA isolation were obtained after a 12 h fasting period. The study protocol was approved by the Ethics Committee at Kunming Institute of Zoology, Chinese Academy of Sciences. Written informed consent was obtained from each of the participants prior to the study.

### Choice of SNPs, DNA isolation and genotyping

18 reported longevity-associated SNPs were chosen from the GWAS and other literature databases (MEDLINE, EMBASE, Elsevier, Springer, CINAHL, EBSCO, Highwire Press, LWW, ISI Web of Science and Cochrane Library) for the study (Table 3). All SNPs were selected following the criteria: 1) the association of the SNPs or their target genes/proteins with longevity is reported by at least 1 independent study; 2) the SNPs were either C/T or A/G which is for being compatible with the genotyping system used (Beckman Coulter, Fullerton, CA, USA); and 3) SNPs located no matter where they are (coding gene, outside or in intronic regions). Total genomic DNA was isolated from peripheral EDTA blood samples using a standard phenol/chloroform method [31]. Multiplex polymerase chain reaction (PCR) and SNP analyses were performed using the GenomeLab SNPstream Genotyping System (Beck-man Coulter, Fullerton, CA) following the manufacturers' protocols as described by Ana et al. [32]. All of the A/G genotypes were transformed into C/T genotypes for analysis. Samples which were not genotyped successfully were excluded from subsequent analysis. Primers were optimally designed using Web-based software provided by Beckman Coulter (available at http://www.autoprimer.com).

### Statistical analysis

The calculation of genotype and allele frequencies, HWE and further genotypic association were performed using SNPstats (http://bioinfo.iconcologia.net/snpstats/start.htm). Odds ratios (ORs) and respective 95% confidence intervals (95% CI) were used to evaluate the effects of any difference between alleles or genotypes. Allelic association was analyzed using SPSS for Windows software package version 13.0 (SPSS, Inc., Chicago, IL). Differences of < 0.05 were considered significant. Genotypic association was adjusted for sex using four genetic models (codominant, dominant, recessive, and log-additive) and the Akaike information criterion (AIC) was used to choose the genetic model that best fits the data.

## SUPPLEMENTAl TABLES


