# Supplementary material for: Association of the insulin-like growth factor binding protein 3 (IGFBP-3) polymorphism with longevity in Chinese nonagenarians and centenarians
Source: Aging (Albany NY). 2014 Dec 2;6(11):944–51. doi: 10.18632/aging.100703 (PMC4276788; doi:10.18632/aging.100703)
Supplement: Supplementary file 1 [file aging-06-944-s001.pdf]

32. Valdes AM, Van Oene M, Hart DJ, Surdulescu GL, Loughlin J, Doherty M and Spector TD. Reproducible genetic associations between candidate genes and clinical knee osteoarthritis in men and women. Arthritis and rheumatism. 2006; 54:533-539.

SUPPLEMENTAL TABLES

Supplemental Table 1. Allelic distributions of selected SNPs in general Chinese Han and longevity subjects.

	Control		Control	Longevity		Longevity	Allelic analysis			
	Major allele	Minor allele	number	Major allele	Minor allele	number	χ2	OR	%95 CI	*p* Value
rs2717536[a]	297 (0.71)	119 (0.29)	208	832 (0.73)	314 (0.27)	573	0.222	0.942	0.734~1.208	0.655
rs2153960	217 (0.80)	57 (0.20)	274	800 (0.70)	346 (0.30)	573	5.665	1.367	1.056~0.769	**0.02**
rs1377638	154 (0.56)	120 (0.44)	274	647 (0.56)	499 (0.44)	573	0.006	0.99	0.759~1.291	0.946
rs10069397	255 (0.93)	19 (0.07)	274	1056 (0.92)	90 (0.08)	573	0.264	1.144	0.685~1.911	0.705
rs1245541	244 (0.90)	26 (0.10)	270	980 (0.86)	166 (0.14)	573	4.395	1.59	1.027~2.460	**0.038**
rs2244621	138 (0.50)	136 (0.50)	274	568 (0.50)	578 (0.50)	573	0	0.997	0.766~1.298	0.518
rs11977526	229 (0.84)	43 (0.16)	272	887 (0.77)	259 (0.23)	573	6.049	1.555	1.092~2.215	**0.008**
rs1063192	215 (0.80)	55 (0.20)	270	942 (0.82)	204 (0.18)	573	0	1	0.034~29.807	0.8
rs579327	260 (0.95)	14 (0.05)	274	1033 (0.90)	113 (0.10)	573	6.129	2.032	1.147~3.599	**0.013**
rs1455311	225 (0.82)	49 (0.18)	274	958 (0.84)	188 (0.16	573	0.014	1.021	0.724~1.440	0.491
rs13217795[a]	317 (0.77)	94 (0.23)	208	789 (0.70)	345 (0.30)	567	8.459	1.175	1.134~1.918	**0.002**
rs2219078	188 (0.69)	86 (0.31)	274	758 (0.67)	376 (0.33)	567	0.314	1.084	0.817~1.440	0.616
rs2755213	163 (0.60)	111 (0.40)	274	680 (0.60)	454 (0.4)	567	0.021	0.98	0.749~1.283	0.891
rs12629971	184 (0.67)	90 (0.33)	274	716 (0.63)	418 (0.37)	567	1.542	1.194	0.903~1.578	0.234
rs1003533	181 (0.66)	93 (0.34)	274	684 (0.61)	446 (0.39)	567	2.849	1.269	0.962~1.674	0.097
rs189037	202 (0.52)	190 (0.48)	196	662 (0.59)	468 (0.41)	567	5.9	0.752	0.597~0.947	**0.009**
rs1442709[b]	147 (0.54)	127 (0.46)	274	633 (0.56)	501 (0.44)	567	0.421	0.916	0.703~1.194	0.542
rs6817112[a]	135 (0.66)	71 (0.34)	103	734 (0.65)	400 (0.35)	567	0.05	1.036	0.758~1.416	0.874

OR, odds ratio; 95% CI, 95% confidence interval; [a] Data of the control group are from the the 1000 Genomes Project; [b] Data of the control group are from published literature (Li S, et al. Functional polymorphism rs189037 in the promoter region of ATM gene is associated with angiographically characterized coronary stenosis. Atherosclerosis. 2011; 219(2):694-697.); The rest data of control group without annotation are from the Hapmap Project; P-values were not adjusted by sex.

Supplemental Table 2. Genotype association with longevity in Chinese nonagenarians and centenarians.

	Model	Genotype	Control	Longevity	OR (95% CI)	P-value	AIC	BIC
rs2717536		C/C	290 (50.6%)	229 (48.7%)	1			
	Codominant	T/C	252 (44%)	210 (44.7%)	1.04 (0.80-1.34)	0.71	1400.3	1420.1
		T/T	31 (5.4%)	31 (6.6%)	1.25 (0.73-2.15)			
	Dominant	C/C	173 (50.6%)	229 (48.7%)	1	0.6	1398.8	1413.6
		T/C-T/T	169 (49.4%)	241 (51.3%)	1.08 (0.82-1.42)			
	Recessive	C/C-T/C	322 (94.2%)	439 (93.4%)	1	0.66	1398.3	1413.2
		T/T	20 (5.8%)	31 (6.6%)	1.14 (0.64-2.03)			
	Overdominant	C/C-T/T	193 (56.4%)	260 (55.3%)	1	0.75	1399	1413.8
		T/C	149 (43.6%)	210 (44.7%)	1.05 (0.79-1.39)			
	Log-additive	---	---	---	1.07 (0.85-1.35)	0.55	1398.5	1413.4
rs2153960	Codominant	T/T	281 (49%)	233 (49.4%)	1	0.84	1404.6	1424.4
		T/C	238 (41.5%)	201 (42.6%)	1.01 (0.78-1.31)			
		C/C	54 (9.4%)	38 (8.1%)	0.88 (0.56-1.39)			
	Dominant	T/T	281 (49%)	233 (49.4%)	1	0.89	1402.9	1417.8
		T/C-C/C	292 (51%)	239 (50.6%)	0.98 (0.77-1.26)			
	Recessive	T/T-T/C	519 (90.6%)	434 (92%)	1	0.56	1402.6	1417.5
		C/C	54 (9.4%)	38 (8.1%)	0.88 (0.56-1.36)			
	Overdominant	T/T-C/C	335 (58.5%)	271 (57.4%)	1	0.84	1402.9	1417.8
		T/C	238 (41.5%)	201 (42.6%)	1.03 (0.80-1.32)			
	Log-additive	---	---	---	0.97 (0.80-1.17)	0.72	1402.8	1417.7
rs1377638	Codominant	C/C	188 (32.8%)	164 (35%)	1	0.35	1397.8	1417.5
		T/C	271 (47.3%)	226 (48.2%)	0.92 (0.70-1.22)			
		T/T	114 (19.9%)	79 (16.8%)	0.77 (0.53-1.10)			
	Dominant	C/C	188 (32.8%)	164 (35%)	1	0.32	1396.9	1411.7
		T/C-T/T	385 (67.2%)	305 (65%)	0.88 (0.67-1.14)			
	Recessive	C/C-T/C	459 (80.1%)	390 (83.2%)	1	0.18	1396.1	1410.9
		T/T	114 (19.9%)	79 (16.8%)	0.80 (0.58-1.11)			
	Overdominant	C/C-T/T	302 (52.7%)	243 (51.8%)	1	0.93	1397.8	1412.7
		T/C	271 (47.3%)	226 (48.2%)	1.01 (0.79-1.30)			
	Log-additive	---	---	---	0.88 (0.74-1.05)	0.16	1395.9	1410.8
rs10069397	Codominant	C/C	484 (84.5%)	387 (82.7%)	1	0.8	1398.3	1418.1
		T/C	88 (15.4%)	80 (17.1%)	1.10 (0.78-1.54)			
		T/T	1 (0.2%)	1 (0.2%)	1.75 (0.11-28.20)			
	Dominant	C/C	484 (84.5%)	387 (82.7%)	1	0.55	1396.4	1411.2
		T/C-T/T	89 (15.5%)	81 (17.3%)	1.11 (0.79-1.55)			
	Recessive	C/C-T/C	572 (99.8%)	467 (99.8%)	1	0.7	1396.6	1411.4
		T/T	1 (0.2%)	1 (0.2%)	1.73 (0.11-27.78)			

	Overdominant	C/C-T/T	485 (84.6%)	388 (82.9%)	1	0.58	1396.4	1411.3
		T/C	88 (15.4%)	80 (17.1%)	1.10 (0.78-1.54)			
	Log-additive	---	---	---	1.11 (0.80-1.55)	0.53	1396.3	1411.2
rs1245541		C/C	421 (73.5%)	344 (73.3%)	1			
	Codominant	T/C	138 (24.1%)	118 (25.2%)	1.05 (0.78-1.40)	0.74	1400.1	1419.9
		T/T	14 (2.4%)	7 (1.5%)	0.73 (0.29-1.85)			
	Dominant	C/C	421 (73.5%)	344 (73.3%)	1	0.89	1398.7	1413.6
		T/C-T/T	152 (26.5%)	125 (26.6%)	1.02 (0.77-1.35)			
	Recessive	C/C-T/C	559 (97.6%)	462 (98.5%)	1	0.48	1398.2	1413.1
		T/T	14 (2.4%)	7 (1.5%)	0.72 (0.28-1.82)			
	Overdominant	C/C-T/T	435 (75.9%)	351 (74.8%)	1	0.72	1398.6	1413.4
		T/C	138 (24.1%)	118 (25.2%)	1.05 (0.79-1.41)			
	Log-additive	---	---	---	0.99 (0.77-1.27)	0.94	1398.7	1413.6
rs2244621		T/T	137 (23.9%)	123 (26.1%)	1			
	Codominant	T/C	294 (51.3%)	233 (49.5%)	0.83 (0.61-1.13)	0.5	1401.6	1421.4
		C/C	142 (24.8%)	115 (24.4%)	0.89 (0.63-1.27)			
	Dominant	T/T	137 (23.9%)	123 (26.1%)	1	0.28	1399.8	1414.6
		T/C-C/C	436 (76.1%)	348 (73.9%)	0.85 (0.64-1.14)			
	Recessive	T/T-T/C	431 (75.2%)	356 (75.6%)	1	0.94	1401	1415.8
		C/C	142 (24.8%)	115 (24.4%)	1.01 (0.76-1.35)			
	Overdominant	T/T-C/C	279 (48.7%)	238 (50.5%)	1	0.31	1400	1414.8
		T/C	294 (51.3%)	233 (49.5%)	0.88 (0.69-1.13)			
	Log-additive	---	---	---	0.95 (0.79-1.13)	0.53	1400.6	1415.4
rs1063192	Codominant	T/T	384 (67%)	300 (64.1%)	1	0.62	1398.6	1418.4
		T/C	174 (30.4%)	151 (32.3%)	1.08 (0.82-1.41)			
		C/C	15 (2.6%)	17 (3.6%)	1.38 (0.67-2.85)			
	Dominant	T/T	384 (67%)	300 (64.1%)	1	0.47	1397.1	1411.9
		T/C-C/C	189 (33%)	168 (35.9%)	1.10 (0.85-1.43)			
	Recessive	T/T-T/C	558 (97.4%)	451 (96.4%)	1	0.42	1396.9	1411.8
		C/C	15 (2.6%)	17 (3.6%)	1.35 (0.66-2.77)			
	Overdominant	T/T-C/C	399 (69.6%)	317 (67.7%)	1	0.66	1397.4	1412.3
		T/C	174 (30.4%)	151 (32.3%)	1.06 (0.81-1.39)			
	Log-additive	---	---	---	1.11 (0.88-1.39)	0.37	1396.8	1411.7
rs579327	Codominant	T/T	472 (82.4%)	398 (84.3%)	1	0.63	1404	1423.9
		C/T	89 (15.5%)	68 (14.4%)	0.95 (0.67-1.35)			
		C/C	12 (2.1%)	6 (1.3%)	0.63 (0.23-1.71)			

	Dominant	T/T	472 (82.4%)	398 (84.3%)	1	0.6	1402.7	1417.5
		C/T-C/C	101 (17.6%)	74 (15.7%)	0.91 (0.65-1.28)			
	Recessive	T/T-C/T	561 (97.9%)	466 (98.7%)	1	0.36	1402.1	1417
		C/C	12 (2.1%)	6 (1.3%)	0.63 (0.23-1.72)			
	Overdominant	T/T-C/C	484 (84.5%)	404 (85.6%)	1	0.82	1402.9	1417.8
		C/T	89 (15.5%)	68 (14.4%)	0.96 (0.68-1.36)			
	Log-additive	---	---	---	0.90 (0.67-1.20)	0.46	1402.4	1417.3
rs1455311		T/T	401 (70%)	327 (69.3%)	1			
	Codominant	T/C	156 (27.2%)	125 (26.5%)	0.97 (0.73-1.28)	0.32	1402.7	1422.5
		C/C	16 (2.8%)	20 (4.2%)	1.66 (0.84-3.30)			
	Dominant	T/T	401 (70%)	327 (69.3%)	1	0.83	1402.9	1417.8
		T/C-C/C	172 (30%)	145 (30.7%)	1.03 (0.79-1.35)			
	Recessive	T/T-T/C	557 (97.2%)	452 (95.8%)	1	0.14	1400.8	1415.6
		C/C	16 (2.8%)	20 (4.2%)	1.67 (0.85-3.31)			
	Overdominant	T/T-C/C	417 (72.8%)	347 (73.5%)	1	0.69	1402.8	1417.7
		T/C	156 (27.2%)	125 (26.5%)	0.94 (0.71-1.25)			
	Log-additive	---	---	---	1.08 (0.86-1.36)	0.5	1402.5	1417.4
rs2219078	Codominant	T/T	249 (43.9%)	221 (43.8%)	1	0.66	1451	1470.9
		T/C	260 (45.9%)	226 (44.8%)	0.93 (0.72-1.20)			
		C/C	58 (10.2%)	58 (11.5%)	1.12 (0.74-1.69)			
	Dominant	T/T	249 (43.9%)	221 (43.8%)	1	0.76	1449.8	1464.7
		T/C-C/C	318 (56.1%)	284 (56.2%)	0.96 (0.75-1.23)			
	Recessive	T/T-T/C	509 (89.8%)	447 (88.5%)	1	0.46	1449.3	1464.3
		C/C	58 (10.2%)	58 (11.5%)	1.16 (0.78-1.71)			
	Overdominant	T/T-C/C	307 (54.1%)	279 (55.2%)	1	0.45	1449.3	1464.2
		T/C	260 (45.9%)	226 (44.8%)	0.91 (0.71-1.16)			
	Log-additive	---	---	---	1.01 (0.84-1.22)	0.91	1449.9	1464.8
rs2755213	Codominant	C/C	206 (36.3%)	181 (35.8%)	1	0.42	1450.1	1470
		T/C	268 (47.3%)	225 (44.5%)	0.92 (0.70-1.21)			
		T/T	93 (16.4%)	99 (19.6%)	1.16 (0.81-1.65)			
	Dominant	C/C	206 (36.3%)	181 (35.8%)	1	0.88	1449.9	1464.8
		T/C-T/T	361 (63.7%)	324 (64.2%)	0.98 (0.76-1.27)			

	Recessive	C/C-T/C	474 (83.6%)	406 (80.4%)	1	0.24	1448.5	1463.4
		T/T	93 (16.4%)	99 (19.6%)	1.21 (0.88-1.67)			
	Overdominant	C/C-T/T	299 (52.7%)	280 (55.5%)	1	0.29	1448.8	1463.7
		T/C	268 (47.3%)	225 (44.5%)	0.88 (0.69-1.12)			
	Log-additive	---	---	---	1.05 (0.88-1.24)	0.59	1449.6	1464.5
rs12629971		C/C	232 (40.9%)	212 (41.8%)	1			
	Codominant	T/C	252 (44.4%)	222 (43.8%)	0.99 (0.76-1.29)	0.99	1455.7	1475.6
		T/T	83 (14.6%)	73 (14.4%)	1.01 (0.70-1.47)			
	Dominant	C/C	232 (40.9%)	212 (41.8%)	1	0.96	1453.7	1468.6
		T/C-T/T	335 (59.1%)	295 (58.2%)	0.99 (0.78-1.27)			
	Recessive	C/C-T/C	484 (85.4%)	434 (85.6%)	1	0.93	1453.7	1468.6
		T/T	83 (14.6%)	73 (14.4%)	1.02 (0.72-1.44)			
	Overdominant	C/C-T/T	315 (55.6%)	285 (56.2%)	1	0.91	1453.7	1468.6
		T/C	252 (44.4%)	222 (43.8%)	0.99 (0.77-1.26)			
	Log-additive	---	---	---	1.00 (0.84-1.19)	0.99	1453.7	1468.6
rs1003533		C/C	205 (36.3%)	199 (39.5%)	1			
	Codominant	T/C	274 (48.5%)	232 (46%)	0.89 (0.68-1.16)	0.69	1447.4	1467.3
		T/T	86 (15.2%)	73 (14.5%)	0.92 (0.63-1.33)			
	Dominant	C/C	205 (36.3%)	199 (39.5%)	1	0.4	1445.4	1460.3
		T/C-T/T	360 (63.7%)	305 (60.5%)	0.90 (0.70-1.15)			
	Recessive	C/C-T/C	479 (84.8%)	431 (85.5%)	1	0.9	1446.1	1461
		T/T	86 (15.2%)	73 (14.5%)	0.98 (0.69-1.38)			
	Overdominant	C/C-T/T	291 (51.5%)	272 (54%)	1	0.46	1445.6	1460.5
		T/C	274 (48.5%)	232 (46%)	0.91 (0.71-1.17)			
	Log-additive	---	---	---	0.94 (0.79-1.13)	0.51	1445.7	1460.6
rs1442709	Codominant	C/C	186 (32.8%)	164 (32.4%)	1	1	1453.8	1473.7
		T/C	261 (46%)	234 (46.2%)	1.01 (0.76-1.33)			
		T/T	120 (21.2%)	108 (21.3%)	1.00 (0.71-1.40)			
	Dominant	C/C	186 (32.8%)	164 (32.4%)	1	0.98	1451.8	1466.7
		T/C-T/T	381 (67.2%)	342 (67.6%)	1.00 (0.77-1.30)			
	Recessive	C/C-T/C	447 (78.8%)	398 (78.7%)	1	0.97	1451.8	1466.7
		T/T	120 (21.2%)	108 (21.3%)	0.99 (0.74-1.34)			

	Overdominant	C/C-T/T	306 (54%)	272 (53.8%)	1	0.95	1451.8	1466.7
		T/C	261 (46%)	234 (46.2%)	1.01 (0.79-1.29)			
	Log-additive	---	---	---	1.00 (0.84-1.18)	1	1451.8	1466.7
rs6817112		C/C	126 (22.2%)	115 (22.8%)	1			
	Codominant	T/C	322 (56.8%)	289 (57.3%)	0.97 (0.72-1.32)	0.97	1450.7	1470.6
		T/T	119 (21%)	100 (19.8%)	0.96 (0.66-1.40)			
	Dominant	C/C	126 (22.2%)	115 (22.8%)	1	0.83	1448.7	1463.6
		T/C-T/T	441 (77.8%)	389 (77.2%)	0.97 (0.72-1.30)			
		C/C-T/C	448 (79%)	404 (80.2%)	1			
		T/T	119 (21%)	100 (19.8%)	0.98 (0.72-1.33)			
	Recessive	C/C-T/T	245 (43.2%)	215 (42.7%)	1	0.9	1448.7	1463.7
		T/C	322 (56.8%)	289 (57.3%)	0.99 (0.77-1.27)			
		---	---	---	0.98 (0.81-1.18)			

* P-values were adjusted by sex; OR, Odds ratio; %95 CI, 95% confidence interval; AIC, Akaike information criteria; BIC, Bayesian information criteria

Supplemental Table 3. Sex and age of the control and longevity groups.

	Control (n=508)	Longevity (567)	P-value
Sex (male/female)	289/219	216/351	<0.001
Age (years) *	51.7±5.5	94.1±3.2	<0.001

*age is expressed as mean value ± standard deviation.
